# Emergency department responses to nursing shortages

**DOI:** 10.1186/s12245-024-00628-y

**Published:** 2024-04-05

**Authors:** Nicole R. Hodgson, Richard Kwun, Chad Gorbatkin, Jeanie Davies, Jonathan Fisher, on behalf of the ACEP Emergency Medicine Practice Committee

**Affiliations:** 1 Department of Emergency Medicine, Mayo Clinic Arizona, 5777 E Mayo Blvd, Phoenix, AZ 85054 USA; 2 Department of Emergency Medicine, Swedish Medical Center, Issaquah, WA USA; 3 Department of Emergency Medicine, Madigan Army Medical Center, JBLM, Lakewood, WA USA; 4 American College of Emergency Physicians, Irving, TX USA

**Keywords:** COVID-19, Pandemics, Workforce, Nurses, Hospital emergency service

## Abstract

**Background:**

The COVID-19 pandemic exacerbated the nursing shortage, which is predicted to continue to worsen with significant numbers of nurses planning to retire within the next 5 years. There remains a lack of published information regarding recommended interventions for emergency departments (EDs) facing a sudden nursing shortage.

**Methods:**

We queried emergency department leaders from the American College of Emergency Physicians to examine the impact of nursing shortages on EDs and to gather real-world interventions employed to mitigate the effects of the shortage.

**Results:**

Most respondents (98.5%) reported nursing shortages, with 83.3% describing prolonged shortages lasting more than 12 months, with negative impacts such as misses/near-misses (93.9%) and increasing left without being seen rates (90.9%). ED leaders reported a range of interventions, including operational flow changes, utilizing alternative staff to fill nurse roles, recruitment of new nurses, and retention strategies for existing nurses. They employed temporary and permanent pay increases as well as efforts to improve the ED work environment and techniques to hire new nurses from atypical pipelines.

**Conclusion:**

We report a patchwork of solutions ED leaders utilized which may have variable efficacy among different EDs; personalization is essential when selecting interventions during a sudden nursing shortage.

**Supplementary Information:**

The online version contains supplementary material available at 10.1186/s12245-024-00628-y.

## Introduction

Cyclical nursing shortages, both global and localized to the United States of America, existed prior to the COVID-19 pandemic [1]. The COVID-19 pandemic led to a worsening shortage [2]; the 2022 National Council of State Boards of Nursing’s National Nursing Workforce Survey revealed a loss of 100,000 registered nurses (RNs) and 34,000 licensed practical and vocational nurses (LPNs/LVNs) due to the pandemic between 2020–2022, with an alarming 28% of nurses planning to retire within the next 5 years [3]. Nursing shortages, especially in emergency medicine (EM), where nurses face significant burnout rates [4], are predicted to worsen.

Worsening nursing staff levels correspond with worsening patient satisfaction [5], deterioration of operational metrics [6], and increasing patient mortality [7]. Despite this, literature searches by our American College of Emergency Physicians’ (ACEP) Emergency Medicine Practice Committee (EMPC) revealed a lack of published interventions for emergency departments (EDs) facing a sudden nursing shortage.

We queried national ED leaders to examine the impact of nursing shortages and to obtain information regarding interventions employed to mitigate the effects of these shortages.

## Methods

We gathered information via an electronic collection tool (Appendix 1) from the ACEP Medical Directors (*N* = 450) and EMPC (*N* = 75) listservs to examine the impact of the nursing shortage on EDs and to collect novel solutions and approaches in an open-ended format. Although the listservs mostly consist of physician leaders, a small number of ACEP administrators as well as resident, medical student, physician assistant, and nurse practitioner representatives serve on the committees and are included in the counts. The Mayo Clinic Institutional Review Board provided an exemption from full review. We performed basic statistical analyses for multiple-choice responses (counts, percentages). For free-text responses, one author (NRH) performed content analysis combining similar responses into categories with counts (Appendix 2).

## Results

We present demographics from our 66 respondents in Table 1. All worked in hospital EDs except one, who worked at a freestanding ED.

**Table 1:** Respondent demographics

	Count (%)
Role	
Chair	16 (24.2%)
Vice Chair	5 (7.6%)
Medical Director	28 (42.4%)
Staff Physician	14 (21.2%)
Other^a^	3 (4.5%)
Practice Setting	
Urban	32 (48.5%)
Suburban	26 (39.4%)
Rural	8 (12.1%)
Critical Access Hospital (Yes)	7 (10.6%)
EM Residency Affiliation	
No	38 (57.6%)
Primary Site	21 (31.8%)
Secondary Site	7 (10.6%)
Trauma Designation	
Level I	19 (28.8%)
Level II	14 (21.2%)
Level III	6 (9.1%)
Level IV	4 (6.1%)
Level V	1 (1.5%)
None	22 (33.3%)
Region	
West	13 (19.7%)
Midwest	16 (24.2%)
Northeast	25 (37.9%)
South	10 (15.2%)
International	0 (0.0%)
Other^b^	2 (3.0%)
Visits/Year	
< 10 K	2 (3.0%)
10 K-30 K	8 (12.1%)
30 K-50 K	20 (30.3%)
50 K-70 K	18 (27.3%)
70 K-90 K	12 (18.2)
90 K-110 K	2 (3.0%)
> 110 K	4 (6.1%)
# Licensed Beds	
< 10	4 (6.1%)
10–25	11 (16.7%)
26–50	36 (54.5%)
51–75	11 (16.7%)
76 +	4 (6.1%)

^a^PA, CMO, Director of Quality; ^b^Mid-Atlantic (2)

We report multiple-choice responses in Table 2.

**Table 2:** Staff shortages

	Count (%)
Worsening ED nurse shortages during pandemic (Yes)	65 (98.5%)
Worsening ED physician shortages during pandemic (Yes)	15 (22.7%)
Average Daily % Nursing (100% = full staff, 0% = total deficit)	
0%	2 (3.0%)
25%	21 (31.8%)
50%	15 (22.7%)
75%	25 (37.9%)
100%	2 (3.0%)
No nursing shortage	1 (1.5%)
Length of nursing shortage	
7 days	0 (0.0%)
1 month	1 (1.5%)
6 months	2 (3.0%)
12 months	5 (7.6%)
> 12 months	55 (83.3%)
No nursing shortage	3 (4.5%)
Near Miss/Error related to nursing shortage	
Yes	62 (93.9%)
No	4 (6.1%)
No shortage	0 (0.0%)
Increase in LWBS^a^ during shortage	
Yes	60 (90.9%)
No	5 (7.6%)
No shortage	1 (1.5%)
Boarding in ED worsened compared to pre-pandemic (Yes)	61 (92.4%)
Inpatient staff shortages contributing to boarding (Yes)	60 (90.9%)
Travel nurses used during shortage (Yes)	61 (92.4%)

^a^Left Without Being Seen

We report free-text response summaries in Appendix 2 with numbers of respondents noted as (x#). We describe key results from free-text responses below.

When queried regarding ED interventions to reduce demands on nurse/technician staff or to increase capacity of nurse/technician staff or supply of other ancillary services, ED leaders commonly repurposed higher-paid staff for ED RN roles, such as physicians administering medications, placing intravenous (IV) lines, and discharging patients. Conversely, some respondents utilized lesser-paid workers including paramedics, emergency medical technicians (EMTs), LPNs, non-emergency RNs, and patient care technician staff to perform basic RN duties such as IV access, blood draws, and administering medications, within the scope of what state regulations allowed. One ED placed a scribe in triage to replace the triage RN, although the respondent clarified that although scribes recorded vitals and chief complaints, they couldn’t perform Emergency Severity Index (ESI) scores. Another ED used virtual mental health sitters instead of physical RNs for patients requiring monitoring for behavioral concerns. ED leaders attempted to decrease RN work requirements by decreasing documentation burden, decreasing discharge vitals requirements, and changing IV drip medications to oral, IV push, or intramuscular routes. Operational changes to decrease the impact of RN shortages included employing a physician-in-triage (PIT) or teletriage model alongside lobby-based care such as formal vertical care spaces with chairs for administration of IV medications or dedicated areas for physician waiting room (WR) evaluations, lab draws, and discharges. Two ED leaders reported closing ED sections due to lack of RNs.

We asked what changes were made to maintain patient safety during RN shortages, and respondents highlighted above-mentioned interventions along with attempts to obtain new RN staff. Several mentioned operational changes implemented for patient safety, such as vertical flow, PIT, and assigning more RNs to the enlarging waiting room pool. Two new interventions mentioned in response to this question included calling patients ESI 1/2/3 who left without being seen (LWBS) and increasing physician order entry requirements (for example, decreasing use of verbal orders).

Although some ED leaders reported their systems employed no strategies to retain existing nurses, several described RN retention efforts. Many focused on improving RN pay either through temporary (retention or shift/incentive bonuses, internal higher-paid travel programs, time-limited rate increases to match travelers) or longer-lasting (increasing RN base/hourly rates or creating an RN clinical pay ladder or RVU model) improvements. Some described non-financial interventions including improvements in the work environment such as a twice daily physician-led “medical minute” educational huddle, promoting a team environment, and improving nurse-patient ratios.

Attempts at recruitment of new staff mainly focused on financial-related incentives such as sign-on bonuses, pay increases, referral bonuses, and RN tuition reimbursement. However, some ED leaders increased the ED RN pipeline by hiring new graduates and international RNs, creation of or increasing enrollment in an RN ED residency or hospital-affiliated RN school, hiring RN students as externs, cross-training non-ED RNs, and creation of internal traveler programs.

## Discussion

Most respondents (98.5%) reported nursing shortages, with 83.3% describing prolonged shortages lasting more than 12 months, with negative impacts such as misses/near-misses (93.9%) and increasing LWBS (90.9%). The shortage impacts both the ED as well as inpatient nurse availability, with 90.9% of respondents attesting that inpatient shortages contributed to boarding in the ED. Most respondents used travel nurses (92.4%), which can be costly to hospital systems, dissatisfying for employed RNs, and potentially harmful to patient safety [8].

Published literature lacks recommendations for best practices during ED RN shortages. Some hospital systems tried to mitigate the crisis through nurse retention efforts and attempts at hiring new nurses, often by improving reimbursement, which may be beyond the abilities of hospital systems or, depending on employment model, outside the purview of ED directors. Some created RN educational programs such as ED RN residencies or student rotations; however, those solutions take time and may not be beneficial in an acute crisis. Similarly, cross-training RNs for float pools or internal traveler programs takes initial investment. Previous publications support boosting retention by improving the workplace environment and enhancing support systems for ED RNs [9]; our survey respondents reported implementing a physician-led “medical minute” and promoting a team atmosphere. Building RN mentoring programs and implementing other changes to improve the ED environment may be rapidly accessible by an ED director working closely with nurse leadership and may improve retention rates.

Additional interventions included use of alternative staff to offload RN duties. ED directors should consider individual aspects of their EDs and state regulations prior to experimenting with these changes, as some options considered beneficial at one hospital were not felt to be helpful at others. The practice parameters of alternative staff replacing RNs must be clearly established to be successful in the ED. Though lesser-trained than RNs for emergency medicine departmental work, paramedics completing the National Standard Paramedic Curriculum meet or exceed 90% of knowledge, skills and competencies for Certified Emergency Nurse and Critical Care Registered Nurse board certification [10]. Non-traditional staff in triage may increase wait times and LWBS rates [11], although when incorporated into the ED as complementary team members, EMTs and LPNs have helped decrease length of stay (LOS) [12].

Multiple respondents reported operational changes to improve flow and strive for safe care, which may include accelerated diagnostic pathways, fast track, team triage, and technological enhancements of existing processes, alongside alternative space utilization for clinical care. Although suboptimal, the WR may be the only space available to initiate care [13, 14]. WR assessments do not overcome nurse-driven rate-limiting steps but can facilitate diagnostic and therapeutic actions not requiring time-intensive bedside tasks. WR care overlaps with dedicated PIT models, which in certain circumstances can identify and accelerate rate-limiting steps such as advanced imaging or time-sensitive treatment such as early antibiotics or thrombolytics. The rise of WR care may be a symptom of unsafe and overcrowded department conditions, but when used strategically if physician and nurse staffing permits, it can decompress bottlenecks and provide a secondary layer of safety when the department and triage are overrun [13].

Our study is small and therefore suffers limitations. Response and selection bias may impact our results, as we did experience a low response rate (12.6%). Our study was designed to obtain ED physician leader responses and therefore lacks the perspective of RN leadership. There is a possibility of the same hospital system responding multiple times, as several physicians from the same institution could be contained within the listservs; however, this appears unlikely due to variation in responses. Although our sample size was low, we believe the description of interventions employed will benefit ED directors facing acute nursing shortages. We are unable to isolate the individual impact of each intervention due to our study format and the fact that EDs typically employed combinations of interventions; our report may serve as a launching pad for future research but is only a beginning.

## Conclusion

ED leaders reported a variety of interventions, typically employing multiple methods simultaneously; departmental factors should be considered when selecting interventions at the individual ED level. Future research should investigate the impact of isolated interventions to delineate the most beneficial strategies.

### Supplementary Information

**Supplementary Material 1.**

**Supplementary Material 2.**

## Data Availability

No datasets were generated or analysed during the current study.

## Abbreviations

EDs: Emergency departments
RNs: Registered nurses
LPNs/LVNs: Licensed practical and vocational nurses
EM: Emergency medicine
ACEP: American College of Emergency Physicians
IV: Intravenous
ESI: Emergency Severity Index
PIT: Physician-in-triage
WR: Waiting room
LWBS: Left without being seen
LOS: Length of stay
