# Supplementary material for: Emergency department responses to nursing shortages
Source: Int J Emerg Med. 2024 Apr 5;17:51. doi: 10.1186/s12245-024-00628-y (PMC10996074; doi:10.1186/s12245-024-00628-y)
Supplement: Supplementary file 1 — Supplementary Material 1. [file 12245_2024_628_MOESM1_ESM.docx]

**Appendix 1: Questions**

Please answer the following questions regarding your main site of employment.

1. What is your role in the emergency department?
   1. Chair
   2. Vice Chair
   3. Medical Director
   4. Staff Physician
   5. Other __________________
2. In what setting do you work?
   1. Hospital Emergency Department
   2. Freestanding Emergency Department
   3. Urgent Care
   4. Other _________________________
3. Which best describes your practice setting?
   1. Urban
   2. Suburban
   3. Rural
4. Is your site at a Critical Access Hospital?
   1. Yes
   2. No
5. Are you affiliated with an emergency medicine residency program?
   1. No
   2. Primary emergency medicine residency site
   3. Secondary emergency medicine residency site
6. What is the trauma designation of your site?
   1. Level I Trauma Center
   2. Level II Trauma Center
   3. Level III Trauma Center
   4. Level IV Trauma Center
   5. Level V Trauma Center
   6. Not a trauma center
7. In which region do you practice?
   1. West
   2. Midwest
   3. Northeast
   4. South
   5. International
   6. Other _______________
8. Approximately how many visits per year does your emergency department service?
   1. <10,000
   2. 10,000-30,000
   3. 30,000-50,000
   4. 50,000-70,000
   5. 70,000-90,000
   6. 90,000-110,000
   7. >110,000
9. How many licensed beds are in your emergency department?
   1. <10
   2. 10-25
   3. 26-50
   4. 51-75
   5. 76+
10. Did your emergency department experience worsening ED nursing shortages during the pandemic?
    1. Yes
    2. No
11. Did your emergency department experience worsening ED physician shortages during the pandemic?
    1. Yes
    2. No
12. On average during your nursing shortage, what was your approximate level of daily nursing deficit? 0% reflects no nurses available, 100% represents full nurse staffing for your emergency department.
    1. 0%
    2. 25%
    3. 50%
    4. 75%
    5. 100%
    6. Did not experience a nursing shortage
13. Approximately how long did your nursing shortage last?
    1. 7 days
    2. 1 month
    3. 6 months
    4. 12 months
    5. >12 months
    6. Did not experience a nursing shortage
14. Has your ED experienced a near miss or error related to nurse staffing issues?
    1. Yes
    2. No
    3. Did not experience a nursing shortage
15. Did your ED experience an increase in patients leaving without being seen during your nurse shortage?
    1. Yes
    2. No
    3. Did not experience a nursing shortage
16. Is ED boarding at your site worse now compared to before the pandemic?
    1. Yes
    2. No
17. Are inpatient staffing shortages contributing to increased boarding in your Emergency Department when compared with times prior to the pandemic?
    1. Yes
    2. No
18. To deal with decreasing nursing resources, did your ED implement effective strategies to reduce demands on nursing/tech staff? Examples might include physician-only discharge, IV push medications in the place of piggyback medications, and decreasing documentation requirements for nursing staff. If so, free text the strategy. If you’re willing to share any implemented protocols, please send to [email redacted]. ________________________________________________________________________________________________________________________
19. To deal with decreasing nursing resources, did your ED implement effective strategies to increase capacity of the nursing/tech staff or supply of other ancillary services? Examples might include expanding scope for ancillary services, using ED pharmacists to replace some nursing tasks, or repurposing a “provider in triage” to perform additional duties. If so, free text the strategy. If you’re willing to share any implemented protocols, please send to [email redacted]. _______________________________________________________________________________________________________________________________
20. Did your emergency department implement changes during your nursing shortage to maintain patient safety? If so, please free text the strategy. If you’re willing to share any implemented protocols, please send to [email redacted]. ______________________________________________________________________________________________________________________________________
21. Were the changes that you made beneficial? If you noted any changes to length of stay or other monitored metrics, please describe. _____________________________________________________________________________________________________________________________________
22. Did you employ any strategies to retain your current nursing staff? Were they successful? Please describe. _________________________________
23. Did you employ travel nurses during your shortage?
    1. Yes
    2. No
24. Did you employ any strategies to recruit additional nursing staff? Were they successful? Please describe. _____________________________________________________
25. Are there any other observations related to staff shortages that you would like to provide to our task force? ____________________________________________________________
