# Supplementary material for: Emergency department responses to nursing shortages
Source: Int J Emerg Med. 2024 Apr 5;17:51. doi: 10.1186/s12245-024-00628-y (PMC10996074; doi:10.1186/s12245-024-00628-y)
Supplement: Supplementary file 2 — Supplementary Material 2. [file 12245_2024_628_MOESM2_ESM.docx]

**Appendix 2: Free-Text Responses**

Free text individual respondent counts - Question 18: 43 respondents; Question 19: 41 respondents; Question 20: 30 respondents; Question 21: 26 respondents; Question 22: 41 respondents; Question 24: 35 respondents; Question 25: 27 respondents

**Question 18 and 19:**

When queried regarding ED interventions to reduce demands on nurse/technician staff or to increase capacity of nurse/technician staff or supply of other ancillary services, ED leaders listed interventions in the following categories:

Use of higher-paid staff for ED RN roles: EDs used physicians to perform RN or technician roles. This included physicians discharging patients (x14), administering medications (x4), placing intravenous lines (IVs) (x2), cleaning rooms (x2), rooming patients and performing transport (x3), performing EKGs (x1), and making or receiving phone calls (x1). Two respondents did not specify the tasks but simply reported “MD perform RN tasks” and “APP [NP or PA]/physician-only management.” One respondent reported NP or PA discharge of patients, and another described a PA replacing the triage RN, with the triage RN removed to work bedside in the main ED. This ED leader described that they provided an $800 stipend for this shift but it was still undesirable, with no physicians and few PAs signing up for the role.

Use of lesser-paid or differently-trained staff for ED RN roles: Respondents reported differently-trained workers performing basic RN duties. Many utilized paramedics or emergency medical technicians (EMTs) (x10), with several mentioning using them specifically for IV starts (x2) or obtaining laboratory studies and administering medications (x3, with 1 clarifying that the EMT was physically located in triage). Two EDs expanded the practice scope of pre-existing ED-based paramedics. Multiple EDs hired new LPNs to offload ED RNs (x7, with 1 specifying this was their first time using them in the ED and 1 clarifying they were allowed to give intramuscular or oral but not IV medications). One respondent hired new Patient Care Technician (PCT) staff. Two ED leaders reported leveraging medical-surgical RNs to perform ED RN duties, including transporting patients (x1) and caring for boarders in the ED (x1). One ED placed a scribe in triage to replace the triage RN, although the respondent clarified that although scribes recorded vitals and chief complaints, they couldn’t perform Emergency Severity Index (ESI) scores. Another ED used virtual mental health sitters.

Attempts to recruit new RNs: Three EDs worked with hospital systems to create in-house staffing pools (one reported this was their first hospital float pool, but another noted this was a limited resource). Two wrote about use of travel RNs in this section, although we note that in the multiple-choice section, 92.4% of EDs employed travel RNs. Some respondents referenced attempts at hiring RNs (x1) or technicians (x2) and implementing nurse/technician education programs to recruit new staff (x1).

Incentivizing existing staff: Although queried specifically about this later in our instrument, several responses in this section referenced RN incentives to encourage extra shifts (x1) and pay incentives (x1). One considered an RVU model for nurses (x1).

Reducing RN work requirements: We received multiple reports of attempts to decrease RN work requirements, from decreasing documentation (x7, 1 specifying stopping chief complaint documentation), to decreasing discharge vitals requirements (x1), to changing IV drips to push medications (x5) or emphasizing oral instead of IV medications (x1).

Operational changes: ED leaders reported altering their ED flow to decrease the impact of RN shortages. Multiple employed a physician-in-triage (PIT) model (x9), although one specified that “our physicians dislike this role” and another discontinued PIT when it worsened ED throughput. One described use of a “teletriage provider.” Respondents commonly employed lobby-based care outside of a PIT model (x6), with some creating a formal vertical care space with chairs for administration of IV fluids and medications and others dedicating an area for physician waiting room (WR) evaluations, lab draws, and discharges. One respondent dedicated an RN to the WR to medicate or discharge patients without taking them on as an official assignment. Two ED leaders reported closing sections of their EDs due to lack of RNs.

**Question 20:**

We asked whether EDs made any changes to maintain patient safety during nursing shortages, and respondents mostly highlighted the above-mentioned interventions: closing ED sections to maintain nursing ratios (x3, 1 noting that Fast Track was closed), system-wide RN float pools to staff EDs (x1), travel RNs (x1, although they highlighted this was a significant cost), hiring technicians (x1) or LPNs (x2) to offload RNs, having MDs perform RN tasks (x1), and replacing the triage RN with a PA (x1). Several mentioned operational changes as having been implemented for patient safety: vertical flow for mid-acuity patients (x1; before this intervention mid-acuity patients experienced 18+ hour waits), implementing PIT (x1), moving RNs from the main ED to the WR to have multiple eyes on a large cohort of patients (x1), and assigning a lobby nurse with a sole role of vitals and reassessment every 2 hours (x1). One ED leader simply stated “monitoring the lobby became a passion.” Two new interventions mentioned in response to this question included calling LWBS patients ESI 1/2/3 (x1) and increasing physician order entry requirements (x1 who clarified that verbal orders led to misses and near misses with inexperienced, borrowed, or new staff).

**Question 21:**

We asked whether the implemented changes were beneficial and received various responses.

Beneficial: expanded paramedic scope (x2), LPNs/technicians to offload RNs (x3: 1 specified LPNs helpful to cover the WR, fast track and boarders in the ED, 1 stated results were mediocre), increasing RN pay and incentives to encourage extra shifts (x1), utilization of a second triage area stationed with a physician (x1, specified that LWBS improved), vertical flow processes (x1, described treating increasing numbers of patients vertically since inception), use of medical-surgical RNs to care for boarders in the ED (x1, described increasing ED RN satisfaction).

Mixed or Minimal Impact: travel nurses (x1), “lobby medicine” (x1, specifying it was necessary due to lack of both beds and nurses, and improved LWBS and discharge disposition time but decreased patient and physician satisfaction), switching to IV push / oral over IV medications (x1), PIT combined with paramedic use (x1, increased length of stay (LOS) but improved LWBS), PIT paired with physician administration of medications and rooming/discharge of patients (x1, “made some things less bad…remained a slow moving catastrophe”), PIT paired with APP/physician-only management (x1, decreased LWBS rates from 5% to 1%), physician-only discharge paired with physicians making phone calls, changing beds and starting IVs (x1, improved LWBS rates but did not influence other metrics), WR vertical care paired with LPNs for intramuscular and oral medications, APP-only discharge, reducing discharge vitals requirements, and moving staff from the main ED to monitor the WR (x1, significantly decreased LWBS and similar metrics such as left without completing treatment and elopement but had no impact on LOS).

Not beneficial: PA as triage RN paired with allowing PAs/physicians access to the Pyxis and physicians placing IVs and discharging patients (x1), physicians performing RN tasks (x2, 1 clarifying that it did improve LWBS rates but came at the cost of physician job satisfaction), PIT paired with physician-only discharge, physician administration of medications, virtual mental health sitters and closing ED zones (x1), PIT paired with LPNs, paramedics, decreasing RN documentation and providing RN pay incentives (x1).

**Question 22:**

Although some ED leaders reported their systems employed no strategies to retain existing nurses (x6) or reported that they were unsure (x1) or that the strategies were unsuccessful (x2), several respondents described RN retention efforts:

Improving work environment: Responses included hosting a physician-led “medical minute” huddle twice daily (x1), promoting a team approach which led travelers to want to remain (x1), and improving nurse-patient ratios (x1).

Improving temporary pay: Interventions included retention bonuses (x8) and shift/incentive bonuses (x7, 1 described increasing rates for last-minute coverage for RN call-outs as successful, 2 unfortunately noted that they offered this too late, 1 expressed concern that RNs planned call-outs to increase bonuses). Two systems offered internal travel programs for existing RNs, with one offering higher pay to float between in-system EDs (however reported difficulties as this brought in RNs unfamiliar with their ED). One increased pay specifically for travelers, whereas another increased the hourly rate of existing RNs to nearly match traveler rates on a 13 week contract basis, helping their RNs feel valued.

Improving ongoing pay: Some implemented permanent changes, by increasing RN base or hourly rates (x12, 1 notes too slowly and not enough), renegotiating the RN contract (x1), or creation of an RN clinical pay ladder (x1, although notes questionable success).

**Question 24:**

Although some reported their systems employed no strategies to recruit new nursing staff (x2) or reported that they were unsure (x2) or that the strategies were unsuccessful (x5), leaders reported multiple strategies to recruit new nursing staff in the following categories:

Reimbursement-related efforts: sign-on bonuses (x6), retention bonuses (x1), pay increases (x4, 1 noting the new pay closely matched travel wages), referral bonuses (x1, notes unsure of success), increased traveler pay (x1), and RN tuition reimbursement (x1, notes seems successful).

Increasing the ED RN pipeline: allowing new graduates to work in the ED (x1), international RN hiring (x1, reports so far so good), creation of RN ED residency (x2, 1 notes accredited by Emergency Nurses Association), increasing spots in an existing RN ED residency for new graduates (x1), creation of an RN school by the hospital system to train and hire new graduates from its own program (x1), creation of nursing student programs (x1), hiring nursing students as externs (x1, notes some success but less efficient and safe), internal traveler programs (x2), cross-training RNs from other departments (x1), constant hiring for increased number of ED RN positions (x1), and shortening ED RN orientation (x1).
